# Managers and Statutory Funds

**Published:** 1914-09-12

**Authors:** 


### LEGAL NOTES FOR INSTITUTIONAL WORKERS.

### Managers and Statutory Funds.

A principle which administrative bodies who have the handling of money have carefully to keep in view is that statutory funds can only be applied to the purposes for which they are destined by statute, and not a shilling of these funds can be spent for any other purpose. The Local Government Board ordered an inquiry into the affairs of a fever hospital which was under the management of the Town Council of Coatbridge. The inquiry included charges against the medical superintendent and the matron of the hospital. The Town Council paid the legal expenses incurred by the medical superintendent and the matron in connection with the inquiry, and resolved to levy a public health general assessment to meet these expenses. A ratepayer brought a case against the Town Council to test the legality of that step, and it has been held that the resolution of the Town Council is *ultra vires* and invalid, for, although there was no suggestion of the Town Councillors using their statutory funds to secure their own personal interests, yet there is no distinction in principle between that and using their funds for the personal interests of another; both courses alike constitute a divergence of statutory funds from their statutory purpose.

